# Decreased cyclooxygenase-2 associated with impaired megakaryopoiesis and thrombopoiesis in primary immune thrombocytopenia

**DOI:** 10.1186/s12967-023-04389-9

**Published:** 2023-08-12

**Authors:** Xibing Zhuang, Pengcheng Xu, Yang Ou, Xia Shao, Ying Li, Yanna Ma, Shanshan Qin, Fanli Hua, Yanxia Zhan, Lili Ji, Tiankui Qiao, Hao Chen, Yunfeng Cheng

**Affiliations:** 1 Department of Hematology, Institute of Clinical Science, Zhongshan Hospital, Fudan University, Shanghai 180 Fenglin Rd, Shanghai, 200032 China; 2 Center for Tumor Diagnosis & Therapy, Jinshan Hospital, Fudan University, Shanghai, 201508 China; 3 Department of Hematology, Zhongshan Hospital Qingpu Branch, Fudan University, Shanghai, 201700 China; 4 Institute of Clinical Science, Zhongshan Hospital, Fudan University, Shanghai, 200032 China; 5 Department of Thoracic Surgery, Zhongshan Hospital Xuhui Branch, Fudan University, Shanghai, 200031 China

**Keywords:** Cyclooxygenase-2, Primary immune thrombocytopenia, Megakaryopoiesis, Thrombopoiesis

## Abstract

**Background:**

Cyclooxygenase (COX)-2 is a rate-limiting enzyme in the biosynthesis of prostanoids, which is mostly inducible by inflammatory cytokines. The participation of COX-2 in the maturation of megakaryocytes has been reported but barely studied in primary immune thrombocytopenia (ITP).

**Methods:**

The expressions of COX-2 and Caspase-1, Caspase-3 and Caspase-3 p17 subunit in platelets from ITP patients and healthy controls (HC), and the expressions of COX-2 and CD41 in bone marrow (BM) of ITP patients were measured and analyzed for correlations. The effects of COX-2 inhibitor on megakaryopoiesis and thrombopoiesis were assessed by in vitro culture of Meg01 cells and murine BM-derived megakaryocytes and in vivo experiments of passive ITP mice.

**Results:**

The expression of COX-2 was decreased and Caspase-1 and Caspase-3 p17 were increased in platelets from ITP patients compared to HC. In platelets from ITP patients, the COX-2 expression was positively correlated with platelet count and negatively correlated to the expression of Caspase-1. In ITP patients BM, the expression of CD41 was positively correlated with the expression of COX-2. COX-2 inhibitor inhibited the count of megakaryocytes and impaired the maturation and platelet production in Meg01 cells and bone marrow-derived megakaryocytes. COX-2 inhibitor aggravated thrombocytopenia and damaged megakaryopoiesis in ITP murine model.

**Conclusion:**

COX-2 plays a vital role in the physiologic and pathologic conditions of ITP by intervening the survival of platelets and impairing the megakaryopoiesis and thrombopoiesis of megakaryocytes.

**Supplementary Information:**

The online version contains supplementary material available at 10.1186/s12967-023-04389-9.

## Introduction

Primary immune thrombocytopenia (ITP) is an acquired autoimmune disease manifested by ecchymosis or spontaneous bleeding in the skin and mucosa. Concepts surrounding the mechanisms of thrombocytopenia in ITP involve a loss of immune tolerance [1, 2]. Although humoral or cell-mediated platelet destruction plays an important role in ITP, impaired megakaryocyte maturation and insufficient platelet production also participate in the pathogenesis of ITP. Studies have found that megakaryogenesis and thrombopoiesis in ITP could be disturbed by anti-platelet antibody-mediated destruction and the direct cytotoxicity by CD8^+^ T cells [3–5]. And the abnormal apoptosis of platelets and megakaryocytes was also observed in ITP patients [6, 7], manifesting as activated Caspase-3, -8 and -9 in megakaryocytes and platelets [8]. Besides, the Caspase-1 activity was also elevated in ITP platelets, which indicated the activation of NLRP3 inflammasome [9]. Based on the insufficient megakaryogenesis and thrombopoiesis, the application of recombinant human thrombopoietin (rhTPO) and TPO-receptor agonists has achieved encouraging results in ITP patients [10]. Therefore, the mechanism of abnormal platelet longevity and megakaryocyte function in ITP deserves further exploration.

Cyclooxygenases (COXs) are the rate-limiting enzymes in the biosynthesis of prostanoids. As the two primary isoforms of COXs, COX-1 and COX-2 are responsible for the production of preferential prostanoids [11] COX-2, a major pro-inflammatory factor, is induced by inflammation [12]. Inhibition of COX by non-steroidal anti-inflammatory drugs (NSAIDs) leads to the suppression of caspase family, then further interfering with the initiation of apoptosis and activation of the inflammasome. Studies also found that COX-2 might be related to the development and differentiation of megakaryocytes and the generation of platelets [13, 14]. In a COX-2-deficient mouse model study, COX-2 was found to be associated with physiological megakaryocyte maturation and pro-platelet formation [15]. In addition, COX-2 is involved in the regulation of bone homeostasis, as the osteogenic potential of mesenchymal cells (MSCs) was decreased in the absence of COX-2 [16]. However, little is known about the relationship between COX-2 and ITP.

Based on the correlation of COX2 with megakaryogenesis and thrombopoiesis, we inferred that COX2 may impact the production and count of platelets in ITP. To prove this conjecture, the expressions and correlations of COX-2 in platelets and bone marrow (BM) of adult ITP patients were examined and analyzed. Furthermore, the effects of decreased COX-2 on megakaryopoiesis and thrombopoiesis were assessed by in vitro culture and in vivo murine models for a better understanding of the role of COX-2 in the pathogenesis of ITP.

## Materials and methods

### Patients and healthy controls

Thirty-one newly diagnosed ITP patients who haven’t received any ITP-related treatment from Zhongshan Hospital and Jinshan Hospital, Fudan University, fulfilled the diagnostic criteria according to current guidelines [17] were recruited with their peripheral blood platelet count lower than 100 × 10^9^/L [18]. Patients with hypertension, diabetes, pregnancy, inflammation and other autoimmune diseases or patients who received any kind of nonsteroidal anti-inflammatory drugs within three months prior to enrollment were excluded. Clinical information was also collected including gender, age, platelet count, and treatment history. Thirty-four age- and gender-matched volunteers were served as healthy controls (HC). Formalin-fixed paraffin-embedded (FFPE) bone marrow specimens of 20 primary ITP patients were obtained from Jinshan Hospital pathology archives. The consort diagram for enrolled ITP patients was shown in Additional file 1: Fig. S1. Written informed consents were obtained from each of the participants upon enrollment. The study was approved by the Institutional Review Board of Zhongshan Hospital and Jinshan Hospital, Fudan University, and was conducted in accordance with the Declaration of Helsinki.

### Blood sample preparation

Blood samples were collected from patients with ITP prior to the initiation of any type of treatment. Ten milliliter peripheral blood of each participant was collected with EDTA -anticoagulant vacuum blood collection tube and proceeded to the next step within 15 min. Platelet-rich plasma (PRP) was obtained from anticoagulated blood by centrifugation at 150*g* for 12 min at room temperature with minimum acceleration and deceleration. PRP was carefully removed to a new 15 ml tube with 1/10 volume of acid-citrate dextrose (ACD, 2.5% trisodium citrate, 2.0% D-glucose, 1.5% citric acid) and centrifugated at 900*g* for 15 min at room temperature to isolate platelets [19], the residual blood cells were prepared as leukocytes by lysis of red blood cells. After centrifugation, isolated platelets were washed twice with CGS buffer (123 mM NaCl, 33 mM D-glucose, 13 mM trisodium citrate, pH 6.5) for further analysis [19, 20]. The residual leukocytes in washed platelets were detected by flow cytometry and quantitative real time PCR (qRT-PCR), the leucocytes were set as positive controls in qRT-PCR (Additional file 1: Fig. S2 and S3).

### Murine ITP models

To establish passive murine ITP models, C57BL/6 mice were randomly divided into the control group and the COX-2 inhibitor group. A total of 20 μl peripheral blood was collected from the femoral vein and assessed as untreated platelet count. Mice in the COX-2 inhibitor group were pretreated by intragastric administration of celecoxib (Pfizer Inc., NY, USA) at the dosage of 50 mg/ kg/ d for six days, and mice in the control group received an equivalent volume of normal saline by gavage. Platelet count was assessed after six days of gavage treatment and noted as treated platelet count, then all mice received the anti-mouse CD41 monoclonal antibody (clone MWReg30; BD Biosciences, USA) injection to establish passive ITP models at an initial dose of 0.3 mg/kg body weight and followed by doses of 0.1 mg/kg every 36 h according to previous studies [21, 22]. Mice were continued to be treated by gavage as described above during modeling. Peripheral platelet counts of the femoral vein of mice were monitored every other day. All mice were sacrificed after 7 days, and the femurs were fixed with 4% paraformaldehyde (Yeason, Shanghai, China) or prepared in single-cell suspension for the following experiments.

### Cell culture

Meg01 cell line (Daixuan, Shanghai, China) was incubated in an IMDM medium (Gibco, New York, USA) with 1% penicillin/streptomycin (Gibco, New York, USA) and 20% heat-inactivated fetal bovine serum (Gibco, New York, USA) for the study of megakaryocytes as previous studies in ITP [23, 24]. Based on experimental grouping, PMA-induced Meg01 cells were treated with the selective COX-2 inhibitor celecoxib (Abmole, Houston, TX, USA) at different concentrations (0, 10, 20 and 40 μM) [25, 26] or firocoxib at a concentration of 100 ng/ml in for three days, and then collected for the following experiments.

For the culture of mice bone marrow-derived megakaryocytes, a single cell suspension of bone marrow from wild C57BL/6 mice was collected and cultured in IMDM medium (Gibco, New York, USA) containing 1% penicillin/streptomycin (Gibco, New York, USA) and 20% heat-inactivated fetal bovine serum (Gibco, New York, USA) in the presence of 100 ng/ml recombinant murine thrombopoietin (rmTPO) and 100 ng/ml murine stem cell factor (SCF) for 7 days with or without 10 μM celecoxib. Megakaryocytes were purified and enriched by a 1.5%/3.0% discontinuous bovine serum albumin (BSA) gradient [27] and prepared for the following experiments.

### RNA extraction and qRT-PCR

Total RNA was extracted from megakaryocytes or washed platelets obtained from 14 ITP patients and 14 HCs using Trizol reagent (Life Technology, Carlsbad, California, USA) and reversely transcribed into cDNA with the PrimeScript RT Master Mix (TaKaRa, Dalian, China). The mRNA expression of target genes was quantified by qRT-PCR using the SYBR Premix Ex Taq (Yeason, Shanghai, China). The primer sequences were shown in Additional file 1: Table S1. The relative mRNA expression was calculated as 2^−∆∆ Ct^, the expression of GAPDH was set as control for platelets and actin for megakaryocytes. The Ct values of internal control for all samples are less than 18.

### Protein extraction and Western Blotting

Total platelet protein of megakaryocytes or washed platelets from 17 ITP patients and 20 HCs were extracted, quantified and denatured for western blotting. Anti-human/mouse antibodies (all from Cell Signaling Technology, Danvers, Massachusetts, USA) were used to specifically bind to target proteins and for semi-quantitatively analysis, including COX-2, Caspase-1, Caspase-3, Caspase-3 p17 subunit, GAPDH, GATA1 and β-tubulin. The blots were quantified as gray values via Tanon-4500 Gel Imaging System with GIS ID Analysis Software v4.1.5 (Tanon Science & Technology Co., Ltd., Shanghai, China). Correlations between COX-2, Caspase-1, Caspase-3 p17 subunit expression, and peripheral blood platelet count were analyzed respectively.

### Flow cytometry

Single cell suspensions of Meg01 cells, megakaryocytes, and murine bone marrow were collected and stained with the corresponding antibodies (BioLegend, San Diego, CA, USA) according to the manufacturer’s manuals, detailed information of the fluorescent antibodies is shown in Additional file 1: Table S2. For the DNA staining of cell cycle analysis, cells were incubated with 20 µg/ml Hoechst 33,342 (Abmole, Houston, TX, USA) at 37 °C for 20 min, then washed and stained with CD41 and CD61 antibodies as mentioned. For the staining of platelets, the culture supernatants were collected and centrifuged at low rpm to remove cellular components, then stained with CD41 to identify the platelet granules. All cells were resuspended in 500 μl phosphate buffered solution (PBS) for acquisition and the sample loading time was set to 200 s. The acquisition was performed on a FACS AriII flow cytometer (BD Biosciences, San Jose, CA, USA) and data were analyzed using Flowjo software version 10.0.1. Relative counts of cells were calculated as the counts of the target cells within the loading time.

### Multiplex immunofluorescence (mIF)

Fixated bone marrow specimens were sliced up and prepared for staining. Slides were stained with DAPI (Abcam, Cambridge, UK) or Hoechst 33,342 (Abmole, Houston, TX, USA) for nucleus, CD41 or CD61 antibody for megakaryocytes, GATA1 antibody and COX-2 antibody (all from Cell Signaling Technology, Danvers, MA, USA), and were subsequently treated with fluorescence secondary antibodies (PerkinElmer, Waltham, Massachusetts, USA). For live-cell imaging of Meg01 cells, Meg01 cells were stained with a tubulin-tracker green staining kit for living cells (Beyotime, Shanghai, China) and Hoechst 33,342 (Abmole, Houston, TX, USA) after culture according to the manufacturer’s manual. Fluorescence images of mouse bone marrow and Meg01 cells were taken with an LSM confocal microscope (FV3000, Olympus, Tokyo, Japan). The stained slides of clinical specimens were imaged using the Vectra Polaris 1.0.10. InForm 2.4.8 Image Analysis software (Perkin Elmer) was used for spectral unmixing, cell segmentation, and identification and quantification of cellular subsets. The mean fluorescence intensity (MFI) of COX-2 and CD41 was derived from all the images. CD41 positive cell counts were defined as the number of region area > 200 pixels. Correlations between CD41 MFI/ CD41 positive count and COX-2 MFI in BM were analyzed.

### Statistical Analysis

Data were expressed as mean ± SD or median (range). SPSS 19.0 software (SPSS, Chicago, Illinois, USA) was used for statistical analysis. Normality was assessed by Shapiro–Wilk W test. Student’s *t* test and Wilcoxon rank-sum (Mann–Whitney) test were used for data that fulfilled normal distribution and for those that did not, respectively. Pearson’s correlation analysis was used to study the correlationship between different variables. For the comparation of mRNA and protein expression in enrolled individuals, two-tailed *P* values less than 0.01 were considered statistically significant. For the other analysis, two-tailed *P* values less than 0.05 were considered statistically significant.

## Results

### Patients and healthy controls

The study collected 31 blood samples from newly diagnosed ITP patients and 34 blood samples from HC. Fourteen of these 31 ITP (3 males and 11 females, ranging from 20 to 76 years, median 56.5 years) and 14 of 34 HC (5 males and 9 females, ranging from 23 to 55 years, median 46.5 years) by random assign were used for PCR analyses; 17 ITP patients (7 males and 10 females, ranged from 26 to 83 years, median 53 years) and 20 HC (4 males and 16 females, ranged from 26 to 69 years, median 44 years) by random assign were examined by western blot. mIF was used to detect COX-2 expression in FFPE specimens from 20 ITP patients (7 males and 13 females, ranging from 24 to 82 years, median 59.5 years). Patient characteristics including gender, age, and platelet count were shown in Table 1.

**Table 1:** Patient characteristics

	Study cohort	Test methods	Age (Median, range)	Gender (number, %)	Platelet count (Mean ± SD, × 10^9^/L)
ITP patients	1 (N = 14)	qRT-PCR	56.5 (20–76)	M (3, 21.5%)	45.71 ± 21.57
				F (11, 78.5%)	
	2 (N = 17)	WB	53 (26–83)	M (7, 41.2%)	47.94 ± 30.02
				F (10, 58.8%)	
	3 (N = 20)	mIF	59.5 (24–82)	M (7, 35%)	11.85 ± 8.04
				F (13, 65%)	
Healthy controls	1 (N = 14)	qRT-PCR	46.5 (23–55)	M (3, 21.5%)	255.4 ± 40.8
				F (11, 78.5%)	
	2 (N = 20)	WB	44 (26–69)	M (9, 45%)	252.5 ± 75.5
				F (11, 55%)	

*F* female; *M* male

Cohort 1 were applied for qRT-PCR analyses

Cohort 2 for Western Blot analyses

Cohort 3 for mIF analyses

### The expression of COX-2, Caspase-1, Caspase-3, and Caspase-3-p17 in platelets of ITP patients and healthy controls

**Fig. 1:**
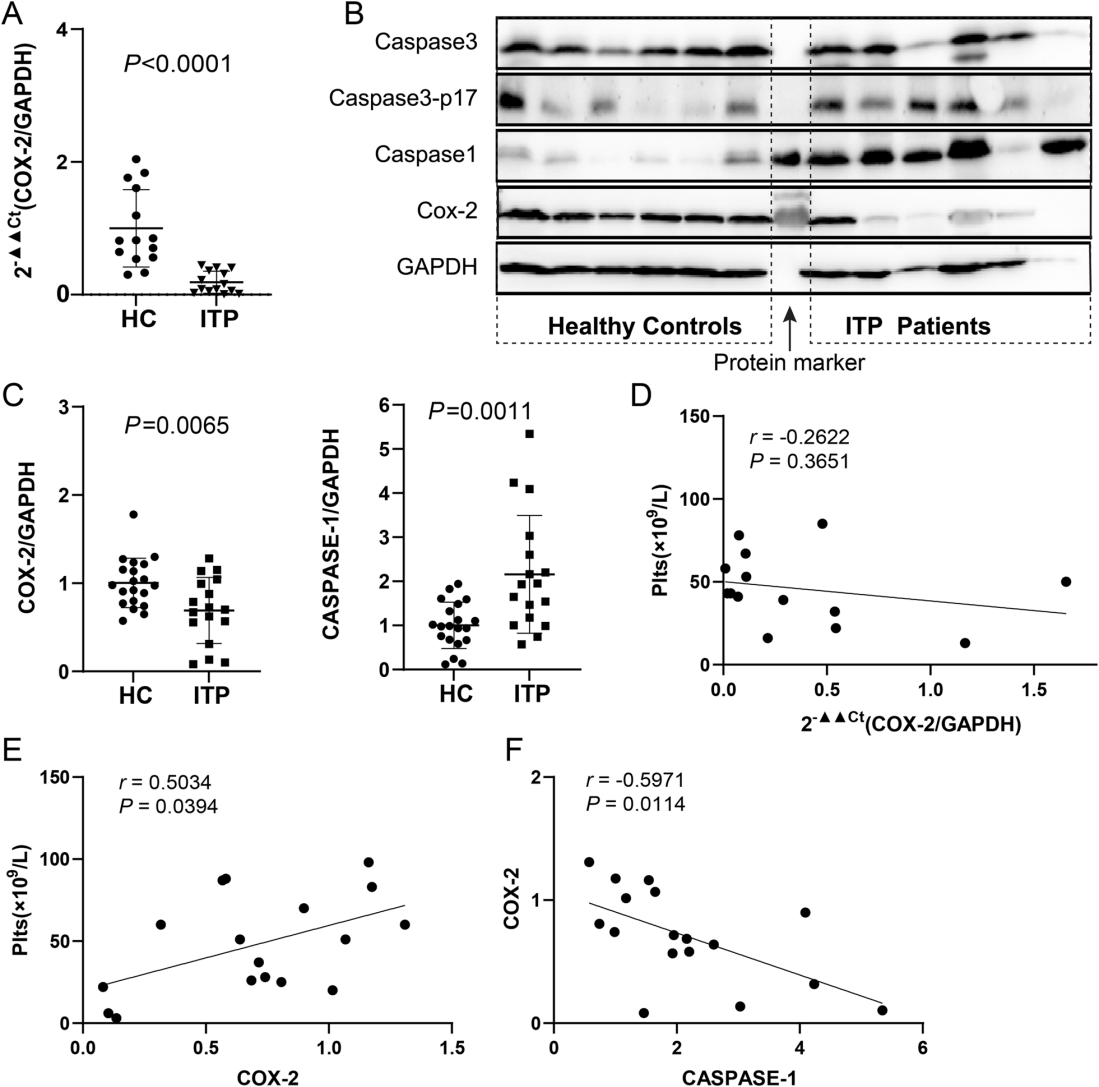
The expressions and correlations of COX-2 and Caspases-1 in platelets of ITP patients and healthy controls. **A** The mRNA levels of COX-2 in platelets. **B** Representative western blot of Caspase-3, Caspase-3 p17 subunit, Caspase-1 and COX-2. **C** Relative protein expression of COX-2 (left) and Caspase-1 (right). Values were calculated as the ratio of the gray values of the target proteins to GAPDH. **D** Correlation of platelet count and mRNA level of COX-2. **E** The correlations of platelet count with the protein expressions of COX-2. **F** The correlations of the expression of COX-2 protein levels with Caspase-1. HC: healthy control, ITP: immune thrombocytopenia, Plts: platelet count

The mRNA levels of COX-2 in platelets were significantly decreased in ITP patients compared to HC (*p* < 0.0001) (Fig. 1A). The protein expressions of COX-2 in platelets were significantly decreased in ITP patients compared to HC (1.003 ± 0.2806 vs. 0.6912 ± 0.3740, *p* = 0.0065), while Caspase-1 was significantly increased (1.002 ± 0.5243 vs. 2.159 ± 1.3360, *p* = 0.0011). The protein levels of Caspase-3 p17 subunit were slightly increased in ITP patients and no significant change was observed in Caspase-3 protein expression between groups (Additional file 1: Fig. S4A, B).

### Correlations among platelet count, COX-2 and Caspase units in platelets of ITP patients

In fourteen ITP patients in cohort 1, there were no correlations between platelet count and COX-2 mRNA levels (Fig. 1D). In 17 ITP patients in cohort 2, the protein expression of COX-2 protein was positively related with platelet count (*r* = 0.5034, *p* = 0.0394) (Fig. 1E) and negatively correlated with Caspase-1 expression (*r* = -0.5971, *p* = 0.0114) (Fig. 1F), but not significantly correlated with Caspase-1, or Caspase-3 p17 subunit expressions (Additional file 1: Fig. S5A–C). These factors were not related to age or gender in ITP patients (Additional file 1: Table S4).

### The expression and correlation of COX-2 on megakaryocytes in bone marrow of ITP patients

**Fig. 2:**
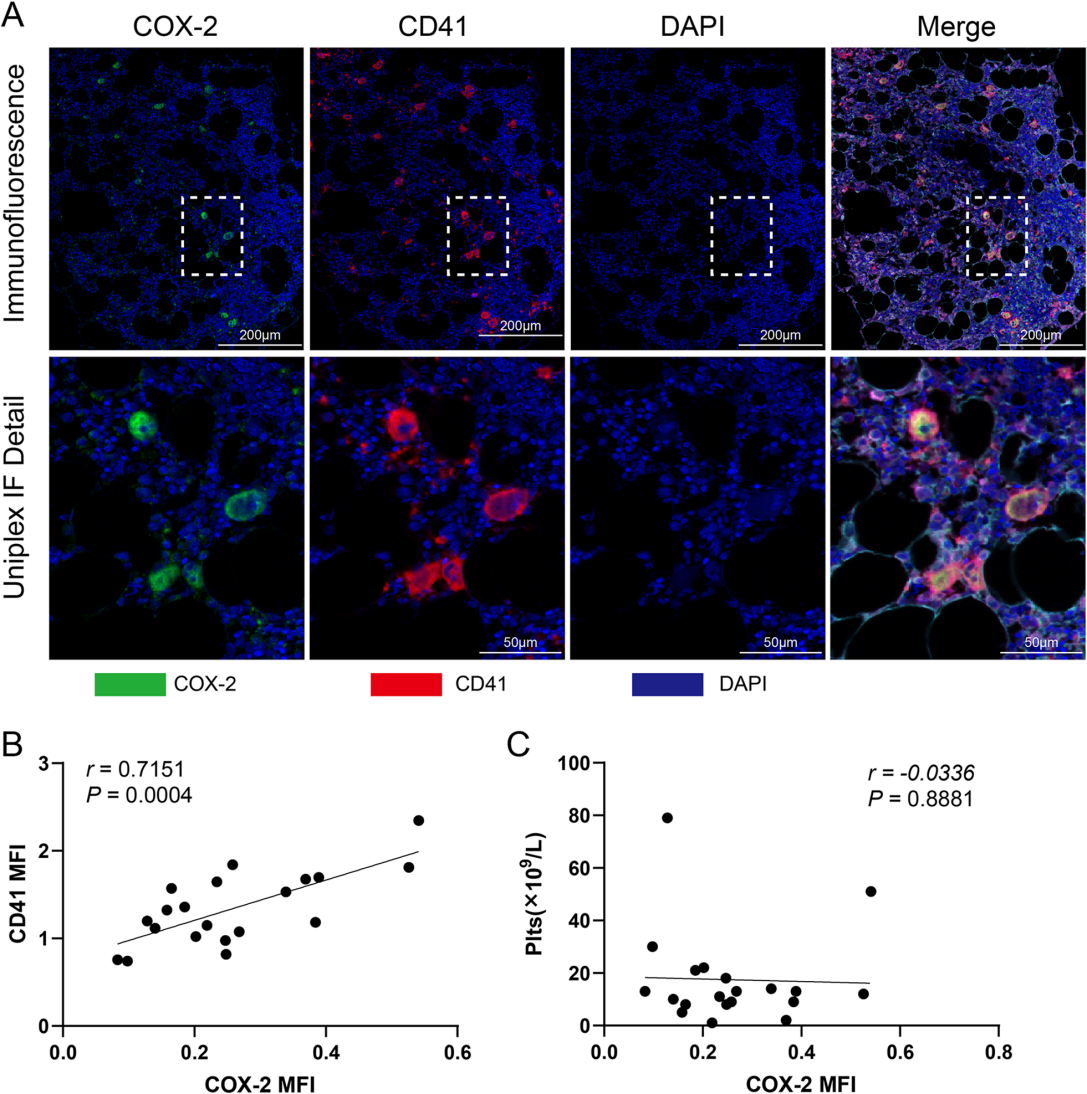
The expressions and correlations of COX-2 on megakaryocytes in bone marrow of ITP patients. **A** Representative multispectral immunofluorescence images of CD41 and COX-2 by multiplex immunofluorescence in BM (magnification × 200, scale bar = 200 μm; magnification × 400, scale bar = 50 μm). Corresponding visualizations of each marker with nuclei (DAPI) for the regions indicated. **B** Correlation between CD41 mean fluorescence intensity (MFI) and COX-2 MFI in CD41 positive cells. **C** Correlation between COX-2 MFI and peripheral platelet count. Plts: platelet count

Representative multispectral immunofluorescence images of CD41 and COX-2 quantification were shown in Fig. 2A. In situ cell protein expressions were quantified by MFI with their correlations analyzed. CD41 MFI was found to be positively correlated with COX-2 MFI in BM (*r* = 0.7151, *p* = 0.0004, Fig. 2B). Yet no significant correlation was observed between COX-2 MFI and platelet count (*p* = 0.8881) (Fig. 2C). No significant correlations were found between total count of megakaryocytes and COX-2 MFI or CD41 MFI (Additional file 1: Table S3). These factors were not related to age or gender in ITP patients (Additional file 1: Table S4).

### Inhibition of COX-2 led to impaired megakaryopoiesis and thrombopoiesis in Meg01 cells

**Fig. 3:**
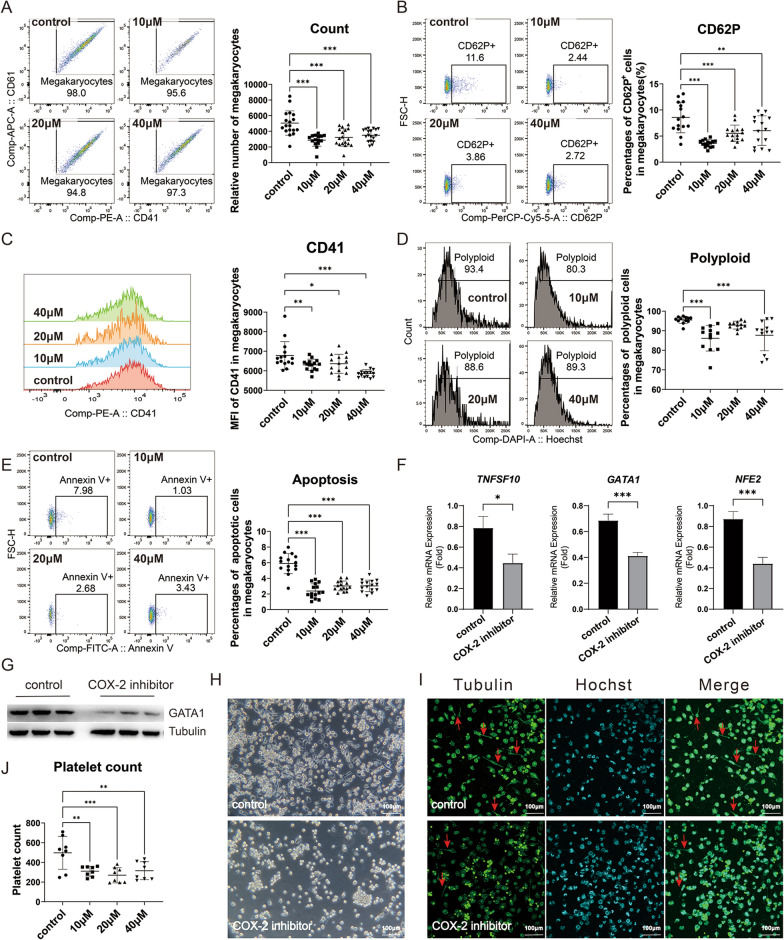
The impacts of COX-2 inhibitor celecoxib on megakaryopoiesis and thrombopoiesis in Meg01 cells. Meg01 cells were treated with different concentrations of COX-2 inhibitor celecoxib in vitro. **A** The relative count of CD41^+^CD61^+^ megakaryocytes in Meg01 cells. Flow cytometry dot plots showed the percentages and gates. **B** Percentages of CD62P^+^ cells in CD41^+^CD61^+^ megakaryocytes. Flow cytometry dot plots showed the percentages and gates for CD62P^+^ cells in CD41^+^CD61^+^ megakaryocytes. **C** Mean fluorescence intensity (MFI) of CD41 in CD41^+^CD61^+^ megakaryocytes. Histogram plots showed the fluorescence intensity of CD41 in flow cytometry. **D** Percentages of polyploid cells in CD41^+^CD61^+^ megakaryocytes. Histogram plots showed the percentages and gates for polyploid cells (N > 4) in CD41^+^CD61^+^ megakaryocytes. **E** Percentages of Annexin V^+^ apoptotic cells in CD41^+^CD61^+^ megakaryocytes. Flow cytometry dot plots showed the percentages and gates for Annexin V^+^ cells in CD41^+^CD61^+^ megakaryocytes. **F** Relative mRNA expression of TNFSF10, GATA1 and NFE2 of Meg01 cells. **G** Western blot plot of the GATA1 expression in Meg01 cells. Tubulin was set as internal reference. **H** Representative light microscopy plot of Meg01 cells without PMA stimulation. **I** Immunofluorescence staining of PMA-stimulated Meg01 cells. Hoechst was used to identify nucleus. **J** Relative count of CD41^+^ platelet granules. *P < 0.05, **P < 0.01, ***P < 0.001

In in vitro culture of Meg01 cells, the selective COX-2 inhibitor celecoxib significantly reduced the relative count of CD41^+^CD61^+^ megakaryocytes at concentrations of 10 μM, 20 μM and 40 μM (Fig. 3A). For the maturation of megakaryocytes, celecoxib significantly decreased the percentages of CD62P^+^ cells (Fig. 3B) and restrained the expression of CD41 in megakaryocytes (Fig. 3C) at concentrations of 10 μM, 20 μM and 40 μM. For the formation of polyploid, celecoxib inhibited the percentages of polyploid megakaryocytes (N > 4) at concentrations of 10 μM and 40 μM (Fig. 3D). For apoptosis, celecoxib withdrawn the percentages of Annexin^+^ apoptotic megakaryocytes at concentrations of 10 μM, 20 μM and 40 μM (Fig. 3E). Since celecoxib showed the demonstrable effects at 10 μM, 10 μM was chosen as the optimal concertation of celecoxib for experiments in vitro. Under this concentration, celecoxib decreased the mRNA levels of megakaryopoiesis related genes (Fig. 3F) and diminished the protein expression of GATA1 (Fig. 3G). In the absence of PMA stimulation, celecoxib reduced the cell attachment and cytoplasmic extensions of Meg01 cells (Fig. 3H). Cytoskeletal tubulin imaging by immunofluorescence showed that after celecoxib treatment, PMA-treated Meg01 cells had fewer cytoplasmic prolongation and tubulin bundles (Fig. 3I). Celecoxib at concentrations of 10 μM, 20 μM and 40 μM resulted in a corresponding reduction of platelet formation in the culture supernatant of Meg01 cells (Fig. 3J). Another selective COX-2 inhibitor, firocoxib, also showed similar effects in Meg01 cells (Additional file 1: Fig. S6).

### Inhibition of COX-2 led to impaired megakaryopoiesis and thrombopoiesis in bone marrow-derived megakaryocytes

**Fig. 4:**
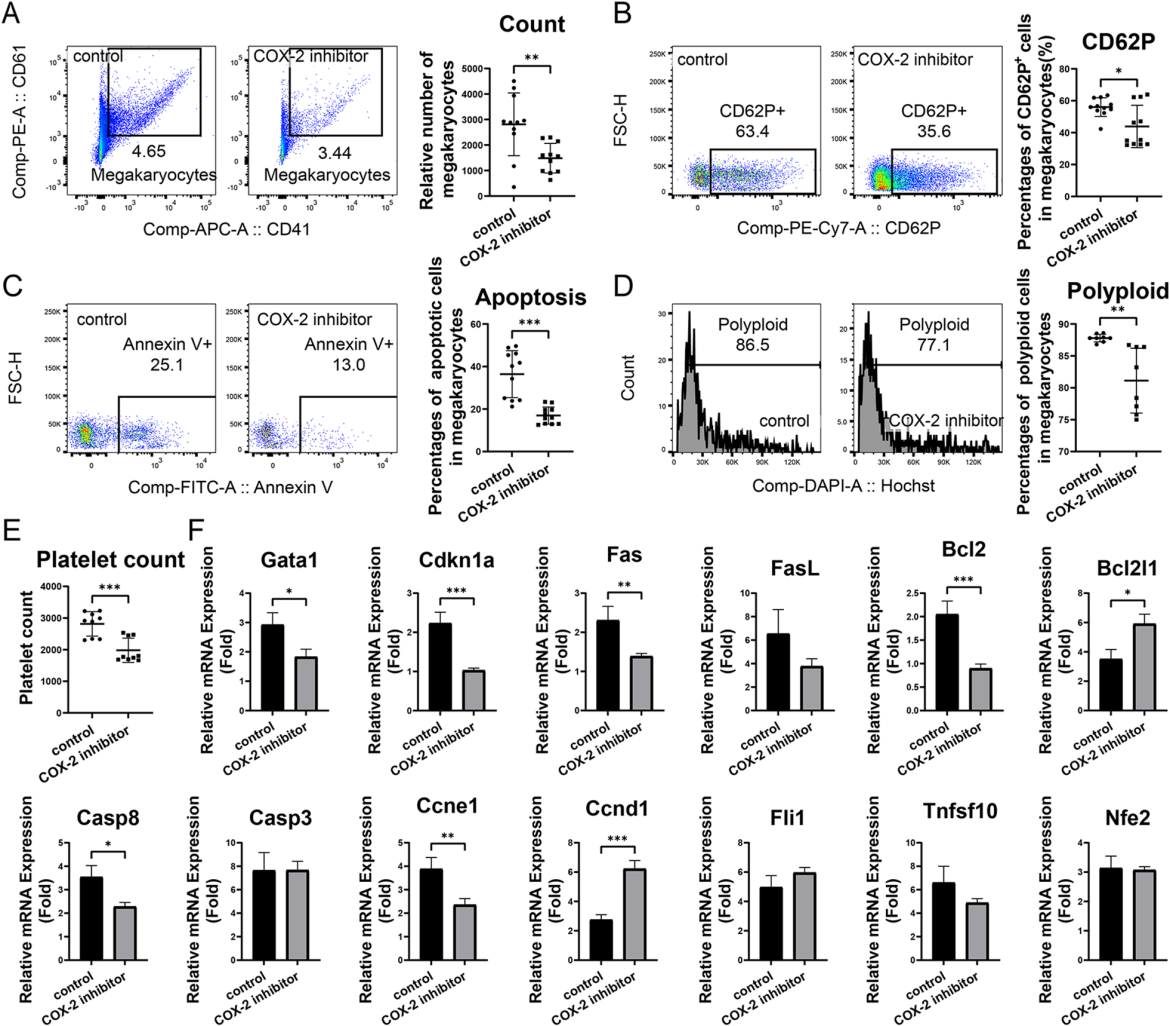
The impacts of COX-2 inhibitor celecoxib on megakaryopoiesis and thrombopoiesis in bone marrow derived megakaryocytes. Single-cell suspension of bone marrow from mice were cultured and induced to differentiate into megakaryocytes under different concentrations of COX-2 inhibitor celecoxib in vitro. **A** The relative count of CD41^+^CD61^+^ megakaryocytes in bone marrow cells. Flow cytometry dot plots showed the percentages and gates for CD41^+^CD61^+^ megakaryocytes in bone marrow cells. **B**, **C** Percentages of CD62P^+^ cells (**B**) and Annexin V^+^ apoptotic cells (**C**) in CD41^+^CD61^+^ megakaryocytes. Flow cytometry dot plots showed the percentages and gates CD41^+^CD61^+^ megakaryocytes. **D** Percentages of polyploid cells in CD41^+^CD61^+^ megakaryocytes. Histogram plots showed the percentages and gates for polyploid cells (N > 4) in CD41^+^CD61^+^ megakaryocytes. **E** Relative count of CD41^+^ platelet granules. **F** Relative mRNA expression of megakaryopoiesis and thrombopoiesis related genes in megakaryocytes. *P < 0.05, **P < 0.01, ***P < 0.001

In in vitro culture of bone marrow-derived megakaryocytes, COX-2 inhibitor celecoxib significantly reduced the relative count of CD41^+^CD61^+^ megakaryocytes (Fig. 4A). For the maturation of megakaryocytes, celecoxib significantly decreased the percentages of CD62P^+^ cells (Fig. 4B). For the apoptosis, celecoxib withdrawn the percentages of Annexin^+^ apoptotic megakaryocytes (Fig. 4C). For the formation of polyploid, celecoxib inhibited the percentages of polyploid megakaryocytes (N > 4) (Fig. 4D). For thrombopoiesis, celecoxib diminished the platelet count in the culture supernatant of mice bone marrow cells (Fig. 4E). Besides, COX-2 inhibitor celecoxib decreased the mRNA levels of megakaryopoiesis related genes such as Gata1and Cdkn1a, and apoptosis related genes Fas and Cas3, decreased mRNA levels of Bcl2 and Ccnd1but increased Bcl2l1 and Ccnd1(Fig. 4F).

### COX-2 inhibitor aggravated the thrombocytopenia and damaged megakaryocytes in ITP murine models

**Fig. 5:**
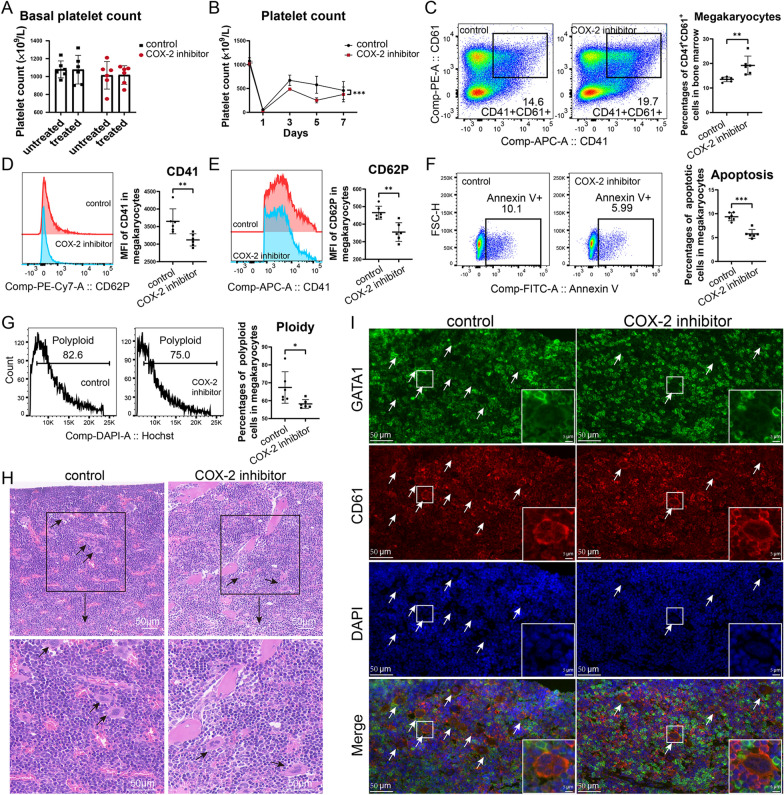
The impacts of COX-2 inhibitor in murine ITP models. Passive ITP murine models were established and treated with or without COX-2 inhibitor celecoxib. (N = 6) **A** Basal platelet count of mice in COX-2 inhibitor group and control group before the establishment of ITP models. Untreated and treated represent the platelet counts of mice before and after the gavage of COX-2 inhibitor or control solvent for six days respectively. **B** Platelet count of mice in COX-2 inhibitor group and control group during the modeling procedure. **C** Percentages of CD41^+^CD61^+^ megakaryocytes in bone marrow cells. Flow cytometry dot plots showed the percentages and gates for CD41^+^CD61^+^ CD41^+^CD61^+^ megakaryocytes. **D** MFI of CD41 on CD41^+^CD61^+^ megakaryocytes. Histogram plots showed the fluorescence intensity of CD41 in flow cytometry. **E**, **F** Percentages of CD62P^+^ cells **E** and Annexin V^+^ apoptotic cells (**F**) in CD41^+^CD61^+^ megakaryocytes. Flow cytometry dot plots showed the percentages and gates in CD41^+^CD61^+^ megakaryocytes. **G** Percentages of polyploid cells in CD41^+^CD61^+^ megakaryocytes. Histogram plots showed the percentages and gates for polyploid cells (N > 4) in CD41^+^CD61^+^ megakaryocytes. **H** HE staining of bone marrow of ITP mice in COX-2 inhibitor group and control group. Plots inside rectangular boxes are magnified and showed below. Arrows marked mature megakaryocytes. **I** Immunofluorescence staining of bone marrow of ITP mice in COX-2 inhibitor group and control group. DAPI was used to identify nucleus. Arrows marked mature megakaryocytes. Megakaryocytes inside rectangular boxes are magnified and showed at the lower right corner of plots. * *P* < 0.05, **P < 0.01, ***P < 0.001

In wild type mice, COX-2 inhibitor celecoxib had no significant influence on platelet count (Fig. 5A), while in the passive murine ITP model, the platelet counts of mice in COX-2 inhibitor group were significantly lower than the control group during the process of model induction (Fig. 5B). Celecoxib increased the percentage of CD41^+^CD61^+^ megakaryocytes in bone marrow (Fig. 5C). The expressions of maturation markers CD41 (Fig. 5D) and CD62P (Fig. 5E) on megakaryocytes were significantly restrained in COX-2 inhibitor-treated mice. The apoptotic megakaryocytes in COX-2 inhibitor treated mice were significantly reduced (Fig. 5F), accompanied by decreased polyploid formation (N > 4) (Fig. 5G). HE staining of bone marrow of ITP mice showed more mature megakaryocytes in bone marrow in the control group compared with the COX-2 inhibitor group (Fig. 5H). Immunofluorescence of the bone marrow showed more CD61 strongly positive megakaryocytes with and higher expression of GATA1 (Fig. 5I).

## Discussion

The pathological mechanisms of ITP remain elusive. Multi-causation mediated platelet destruction and/or decreased platelet production in the pathophysiology of ITP has been universally understood for decades [2, 28, 29]. Specifically, humoral factors and cell-mediated mechanisms, including antiplatelet autoantibodies and defects in T-cell regulation, are involved in platelet clearance and defects in platelet production [30]. In this study, we found the decreased expression of COX-2 in platelets and the positive correlations between COX-2 expression and platelet count or CD41 expression on megakaryocytes in ITP patients. Furthermore, we demonstrated that inhibition of COX-2 by COX-2 inhibitor impaired the megakaryopoiesis and thrombopoiesis of megakaryocytes and led to refractory thrombocytopenia in ITP mice.

COX-1 and COX-2 can catalyze the rate-limiting conversion of arachidonic acid (AA) to pro- and anti-inflammatory prostaglandins and thromboxanes[31]. Compared to COX-1, COX-2 plays a more prominent role in regulating inflammation. It is frequently upregulated and can produce prostaglandins in response to cytokine, phagocytic, or growth factor stimuli in polymorphonuclear leukocytes and monocytes [32]. COX-2 expression plays a crucial role in the regulation and maintenance of immune cells during inflammation, autoimmunity, and lymphoproliferation [33]. Besides, several studies have found that COX-2 was involved in the development of megakaryocytes, for that deficient of COX-2 cause abnormal megakaryopoiesis and platelet function [14, 15]. However, little has been studied about the relationship among platelet counts, the megakaryocyte function and COX-2 expression in ITP patients.

The present study found lower platelet COX-2 expression in adult ITP patients and a positive correlation between COX-2 protein expression and platelet count. These clinical results were hardly reported in ITP but consistent with previous studies beyond ITP, as there has been a reported case of severe drug-induced immune thrombocytopenia (DITP) caused by the selective COX-2 inhibitor etoricoxib [34]. And meta-analyses of COX-2 inhibitors in treating non-small cell lung cancer concluded that COX-2 inhibitors may increase the risk of thrombocytopenia [35–37]. The data suggested that there was no significant correlation between COX-2 mRNA expression and platelet count in ITP, which may be due to the epigenetic and post-transcriptional modifications that may occur in the process of protein synthesis. Nonetheless, the specific role of COX-2 in ITP and the function of COX-2 in platelets and megakaryocytes are still controversial. Peter Rubak et al. examined the COX2 expression in fixed and permeabilized platelets by flow cytometry from five ITP patients and 28 healthy controls [38]. They reported that compared with healthy controls, the COX-2 expression in platelets from whole blood was higher in ITP patients, but no significant difference was found in platelets from PRP [38]. Although adopting the method of separating platelets from PRP similarly, the results from Peter Rubak et al. were not consistent with our present findings. On the one hand, as the authors have mentioned, the limited number of samples and changes in experimental reagents could reduce the stability and sensitivity of their results, on the other hand, platelets from the PRP may lose part of large immature platelets, which may lead to the differences in experimental results [38]. However, our data supported in another way that only newly released platelets express COX-2, which are likely to originate from the cytoplasm of parent megakaryocytes [13]. For all that, the impact of COX2 in platelets in ITP still needs more definitive validation.

CD41 can be considered as a marker of the stage of megakaryocyte differentiation and maturation, and also in platelets. The positive correlation between COX-2 and CD41 in the bone marrow microenvironment revealed that COX-2 plays an important role in the development of megakaryocytes. Although little is reported about COX-2 in megakaryocytes, analogously, a previous study reported a positive correlation between COX-2 and megakaryocytic differentiation induced by diosgenin in human erythroleukemia cells [39], and Tripodo C et al. indicated that COX-2 expression was a feature of myeloid maturation [40]. However, no significant correlation between COX-2 expression in BM and peripheral blood platelets counts was found in adult ITP patients in our results, which can be explained by the fact that the peripheral platelet count of ITP patients depends on multiple factors including increased platelet destruction and/or decreased platelet production. Thus, further experiments about COX2 on megakaryocytes would be necessary to clarify the influences.

To justify the impact of reduced COX2 on megakaryocytes in ITP, we evaluated megakaryocyte maturation and thrombopoiesis both in vivo and in vitro using COX2 inhibitors. Results showed that COX-2 inhibitor impeded the maturation and demolished the platelet production of megakaryocytes both in Meg01 cell line and bone marrow derived megakaryocytes. In ITP mice, COX-2 inhibitor hindered platelet recovery and increased the number of megakaryocytes while blocking their maturation, which is similar to the bone marrow response observed in ITP patients [17]. Comparably, Ivanov capital O, Cyrillic et al. also reported that the predominant blockade of cyclooxygenase-2 increased the number of megakaryocytes in a mouse model, due to the blocking of differentiation into more mature cells [41]. Notably, the administration of an equivalent dose of COX-2 inhibitor in normal mice did not elicit any alterations in platelet count, suggesting the differential effects of COX-2 inhibitor on megakaryocyte function under inflammatory conditions or in the context of specific diseases. Taken together, the in *vivo* and in *vitro* experiments suggested that inhibition of COX-2 is sufficient to damage megakaryopoiesis and thrombopoiesis in ITP and could be the pathogenic factor of ITP.

Caspase is a family of proteases that play an important role in apoptosis. Caspase-1 is a member of the group of caspases which promotes the maturation of interleukin IL-1β and IL-18 by proteolytic cleavage of precursor forms into biologically active proinflammatory cytokines. The Caspase-1 activity was found to be elevated in ITP platelets [9]. Caspase-3 is expressed in cells as an inactive precursor, and the p17 and p11 subunits of the mature Caspase-3 are proteolytically generated during apoptosis. Increased platelet apoptosis was found in a cohort of adult ITP patients, involving loss of mitochondrial membrane potential (ΔΨm), Caspase-3 activation and phosphatidylserine (PS) externalization [42]. Correspondingly, we found the protein expressions of Caspase-1 and Caspase-3 p17 subunits in ITP platelets were higher than that of HCs and negatively correlated with platelet count in ITP patients, which indicated that platelets of ITP were more likely to be destroyed in Caspase-dependent pathway. The anti-inflammation role of NSAIDs has been well known, which selectively blocks Cox-2 activity thus reducing the over-activation of inflammatory mediators [43]. Besides, strong correlations have been reported in several cancer types between COX-2 activity and cell apoptosis [44]. Although studies reported that selective COX-2 inhibitors such as celecoxib could lead to the activation of caspases in tumor cells [45, 46], no significant correlations were found between caspases and COX in ITP platelets, suggesting differences in physiological processes in different cells and different disease background.

The study should be viewed in the light of its limitations. Due to the inaccessibility of bone marrow biopsy specimens of HC, COX-2 expression in BM of ITP patients could not be comparably assessed. Although there were positive results in the correlation analysis, the *r* value was slightly low, which may also be due to the relatively small sample size. Thus, further studies are warranted to investigate the underlying mechanisms of COX-2 in the pathogenesis of ITP.

## Conclusions

This study revealed the reduced expression of COX-2 in platelets in ITP patients, and found correlations between COX-2 expression, platelet count, and maturation of megakaryocytes. Furthermore, the study verified that inhibition of COX-2 impaired the megakaryopoiesis and thrombopoiesis in ITP. Clinically, thrombocytopenia was found in some patients treated with Cox-2 inhibitors, which is also consistent with our study and may provide some theoretical possibilities for us to study the treatment of ITP. These results underscore the important role of COX-2 in platelet production in ITP and provide evidence for COX-2 deficiency as a potential pathogenic factor in ITP.

### Supplementary Information

**Additional file 1:** **Table S1.** Primer sequences of targeted genes. **Table S2.** Fluorescence channels for flow cytometry. **Table S3.** Correlations of CD41, COX-2 and megakaryocytes in bone marrow of ITP. **Table S4.** Correlations of age and gender in ITP patients. **Figure S1.** The consort diagram for enrolled ITP patients. **Figure S2.** The residual leukocytes in washed platelets flow cytometry dot plot showed the percentages of CD45^+^ leukocytes in washed platelets. **Figure S3.** The mRNA levels of residual leukocytes in washed platelets and leukocytes. The qRT-PCR results showed the mRNA expression of CD45 (PTPRC) in washed platelets and leukocytes from ITP patients and healthy controls. Plt: platelets, HC: healthy controls. ^***^P < 0.001. **Figure S4.** The expressions Caspase-3 p17 subunit and Caspases-3 in platelets of ITP patients and healthy controls. **A** Relative protein expression of Caspase-3 p17 subunit in platelets from ITP patients and healthy controls. **B** Relative protein expression of Caspase-3 in platelets from ITP patients and healthy controls. **Figure S5.** The correlations of Caspases, COX-2 and platelet count in ITP patients. **A** The correlations of platelet count with the protein expressions of Caspase-3 p17 subunit. **B** The correlations of platelet count with the protein expressions of Caspase-1. **C** The correlations of the expression of COX-2 protein levels with Caspase-3 p17 subunit. Plts: platelet count. **Figure S6.** The impacts of COX-2 inhibitor firocoxib on megakaryopoiesis and thrombopoiesis in Meg01 cells. Meg01 cells were treated with 100 ng/ml COX-2 inhibitor firocoxib in vitro. **A** The relative count of CD41^+^CD61^+^ megakaryocytes in Meg01 cells. **B** Mean fluorescence intensity (MFI) of CD41 in CD41^+^CD61^+^ megakaryocytes. **C** Percentages of CD62P^+^ cells in CD41^+^CD61^+^ megakaryocytes. **D** Percentages of Annexin V^+^ apoptotic cells in CD41^+^CD61^+^ megakaryocytes. **E** Percentages of polyploid cells in CD41^+^CD61^+^ megakaryocytes. *P < 0.05, **P < 0.01.

## Data Availability

All data generated or analysed during this study are included in this published article (and its additional file).

## Abbreviations

COX-2: Cyclooxygenases -2
ITP: Immune thrombocytopenia
HC: Healthy controls
BM: Bone marrow
MSCs: Mesenchymal cell
IL: Interlukin
BSA: Bovine serum albumin
rmTPO: Recombinant murine thrombopoietin
PRP: Platelet-rich plasma
MFI: Mean Fluorescence intensity
mIF: Multiplex immunofluorescence
FFPE: Formalin-fixed paraffin-embedded
PBS: Phosphate buffered solution
ICH: Intracranial haemorrhage
DITP: Drug-induced immune thrombocytopenia
qRT-PCR: Quantitative real time PCR
PS: Phosphatidylserine
