# Supplementary material for: Decreased cyclooxygenase-2 associated with impaired megakaryopoiesis and thrombopoiesis in primary immune thrombocytopenia
Source: J Transl Med. 2023 Aug 12;21:540. doi: 10.1186/s12967-023-04389-9 (PMC10423426; doi:10.1186/s12967-023-04389-9)

**Table S1: Primer sequences of targeted genes**

| Species | Human | |
| --- | --- | --- |
| Primer name | Primer sequence-Forward | Primer sequence-Reverse |
| GATA1 | CTGTCCCCAATAGTGCTTATGG | GAATAGGCTGCTGAATTGAGGG |
| NFE2 | GCAGGAACAGGGTGATACAG | AATCTGGGTGGATTGAGCAG |
| TNFSF10 | CAGCTCACATAACTGGGACC | CCATTCCTCAAGTGCAAGTTG |
| PTGS2 | TAAGTGCGATTGTACCCGGAC | TTTGTAGCCATAGTCAGCATTGT |
| GAPDH | GAAGGTGAAGGTCGGAGTC | GAAGATGGTGATGGGATTTC |
| PTPRC | ACCACAAGTTTACTAACGCAAGT | TTTGAGGGGGATTCCAGGTAAT |
| ACTIN | CATGTACGTTGCTATCCAGGC | CTCCTTAATGTCACGCACGAT |
| Species | Mice | |
| Primer name | Primer sequence-Forward | Primer sequence-Reverse |
| Gata1 | TGGGGACCTCAGAACCCTTG | GGCTGCATTTGGGGAAGTG |
| Cdkn1a | CCTGGTGATGTCCGACCTG | CCATGAGCGCATCGCAATC |
| Fas | TATCAAGGAGGCCCATTTTGC | TGTTTCCACTTCTAAACCATGCT |
| Fasl | TCCGTGAGTTCACCAACCAAA | GGGGGTTCCCTGTTAAATGGG |
| Bcl2 | GTCGCTACCGTCGTGACTTC | CAGACATGCACCTACCCAGC |
| Bcl2l1 | GACAAGGAGATGCAGGTATTGG | TCCCGTAGAGATCCACAAAAGT |
| Casp3 | ATGGAGAACAACAAAACCTCAGT | TTGCTCCCATGTATGGTCTTTAC |
| Casp8 | TGCTTGGACTACATCCCACAC | TGCAGTCTAGGAAGTTGACCA |
| Fli1 | ATGGACGGGACTATTAAGGAGG | GAAGCAGTCATATCTGCCTTGG |
| Ccnd1 | GCGTACCCTGACACCAATCTC | CTCCTCTTCGCACTTCTGCTC |
| Ccne1 | GTGGCTCCGACCTTTCAGTC | CACAGTCTTGTCAATCTTGGCA |
| Tnfsf10 | ATGGTGATTTGCATAGTGCTCC | GCAAGCAGGGTCTGTTCAAGA |
| Nfe2 | TCCTCAGCAGAACAGGAACAG | GGCTCAAAAGATGTCTCACTTGG |
| Actin | CGTTGACATCCGTAAAGACC | TAGGAGCCAGAGCAGTAATC |

**Table S2: Fluorescence channels for flow cytometry**

| Markers | Fluorescence channels |
| --- | --- |
| Anti-human CD41 | PE |
| Anti-human CD61 | APC |
| Anti-human CD62P | PerCP-CY5.5 |
| Anti-human CD45 | FITC |
| Anti-mice CD41 | APC |
| Anti-mice CD61 | PE |
| Anti-mice CD62P | PE-Cy7 |
| Annexin V | FITC |
| Fiaxble viability stain | BV510 |

**Table S3: Correlations of CD41, COX-2 and megakaryocytes in bone marrow of ITP**

|  |  | Platelet count | COX-2 MFI | CD41 MFI |
| --- | --- | --- | --- | --- |
| CD41 MFI | *r* | 0.087 | 0.715^**^ |  |
|  | *P* | 0.715 | 0.000 |  |
| Total Megakaryocytes (N) | *r* | -0.105 | 0.201 | 0.318 |
|  | *P* | 0.699 | 0.456 | 0.230 |
| Thromocytogenic megakaryocytes (%) | *r* | 0.573^*^ | 0.152 | 0.190 |
|  | *P* | 0.020 | 0.574 | 0.482 |
| Thromocytogenic megakaryocytes (N) | *r* | 0.379 | 0.267 | 0.219 |
|  | *P* | 0.147 | 0.318 | 0.415 |
| Granular megakaryocyte (%) | *r* | -0.599^*^ | -0.163 | -0.172 |
|  | *P* | 0.014 | 0.547 | 0.524 |
| Granular megakaryocyte (N) | *r* | -0.265 | 0.141 | 0.303 |
|  | *P* | 0.321 | 0.602 | 0.254 |
| Promegakaryocytes (%) | *r* | 0.479 | 0.142 | 0.056 |
|  | *P* | 0.060 | 0.600 | 0.837 |
| Promegakaryocytes (N) | *r* | 0.136 | 0.189 | 0.180 |
|  | *P* | 0.614 | 0.484 | 0.505 |

N: number of cells reported in bone marrow cytology, %: percentages of cells in total megakaryocytes. Pearson correlation analysis, ^*^ *P* < 0.05, ^**^ *P* < 0.01.

**Table S4: Correlations of age and gender in ITP patients**

|  |  |  | Age | Gender |
| --- | --- | --- | --- | --- |
| Protein expression | Age | r |  | -0.281 |
|  |  | P |  | 0.274 |
|  | Gender | r | -0.281 |  |
|  |  | P | 0.274 |  |
|  | Platelet count | r | 0.185 | 0.326 |
|  |  | P | 0.478 | 0.202 |
|  | Caspase-1 | r | 0.203 | -0.027 |
|  |  | P | 0.435 | 0.919 |
|  | Caspase -3p17 | r | -0.127 | 0.359 |
|  |  | P | 0.626 | 0.158 |
|  | COX-2 | r | 0.013 | -0.048 |
|  |  | P | 0.960 | 0.854 |
|  | Caspase3 | r | -0.094 | 0.599 |
|  |  | P | 0.719 | 0.611 |
| mRNA expression | Age | r |  | 0.344 |
|  |  | P |  | 0.228 |
|  | Gender | r | 0.344 |  |
|  |  | P | 0.228 |  |
|  | COX-2 | r | 0.119 | 0.241 |
|  |  | P | 0.684 | 0.406 |
|  | Platelet count | r | -0.003 | -0.227 |
|  |  | P | 0.993 | 0.436 |

**Figure S1 The consort diagram for enrolled ITP patients.**

**
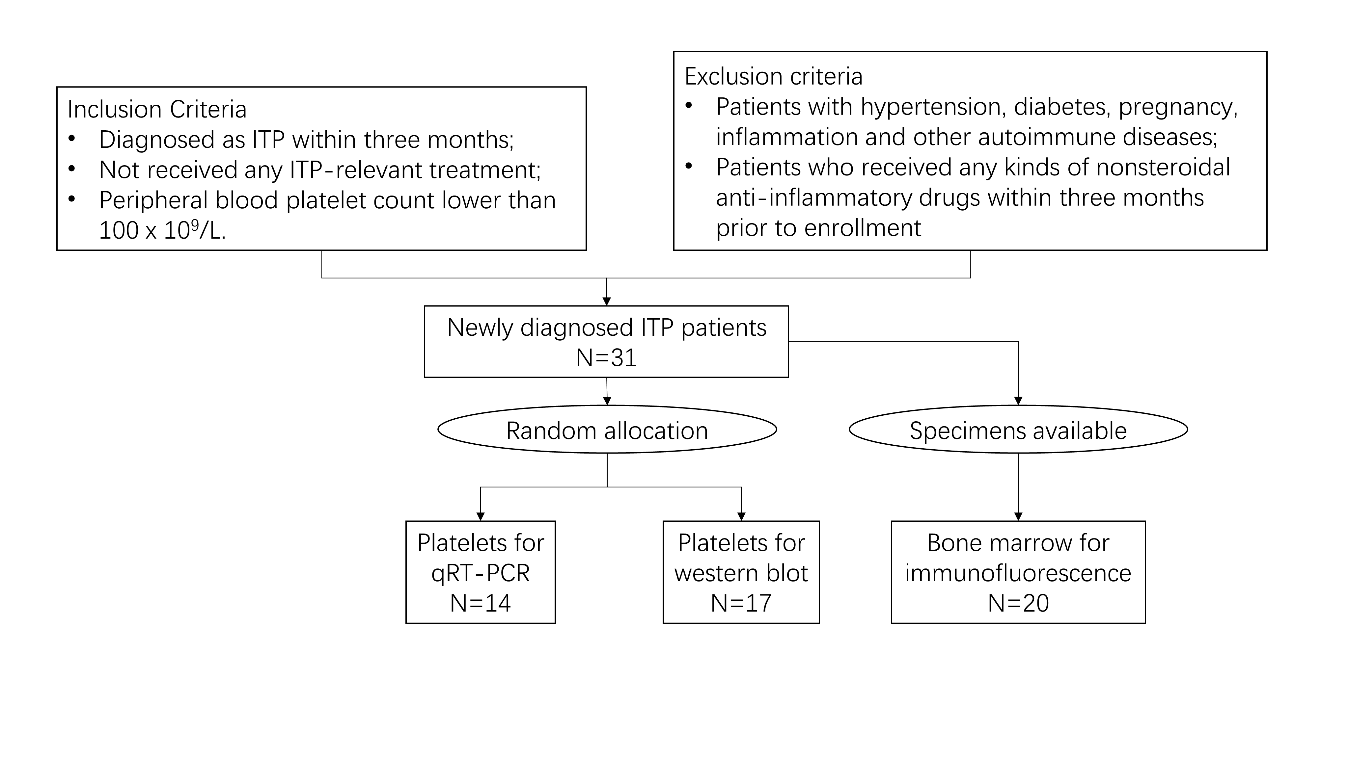
**

**Figure S2 The residual leukocytes in washed platelets.** flow cytometry dot plot showed the percentages of CD45^+^ leukocytes in washed platelets.


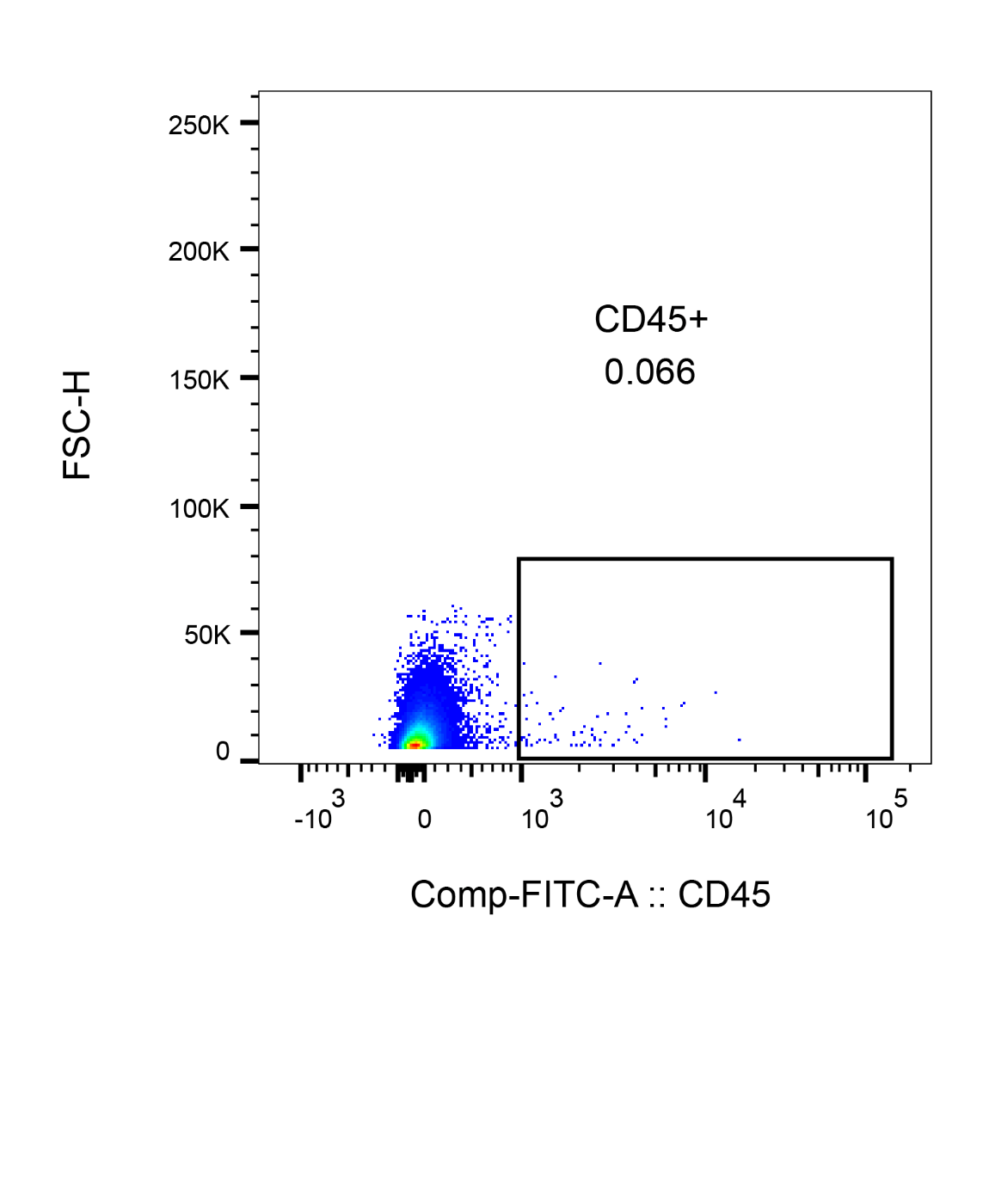


**Figure S3 The mRNA levels of residual leukocytes in washed platelets and leukocytes.** QRT-PCR results showed the mRNA expression of CD45 (PTPRC) in washed platelets and leukocytes from ITP patients and healthy controls. Plt: platelets, HC: healthy controls. ^*^^**^ *P* < 0.001.


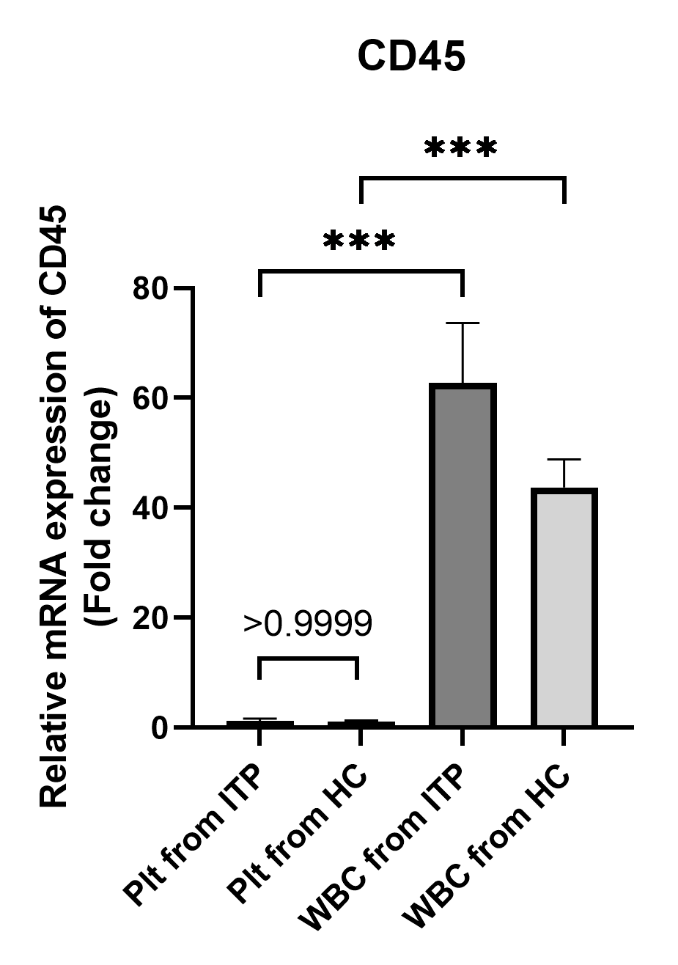


**Figure S4 The expressions Caspase-3 p17 subunit and Caspases-3 in platelets of ITP patients and healthy controls.** (A) Relative protein expression of Caspase-3 p17 subunit in platelets from ITP patients and healthy controls. (B) Relative protein expression of Caspase-3 in platelets from ITP patients and healthy controls.


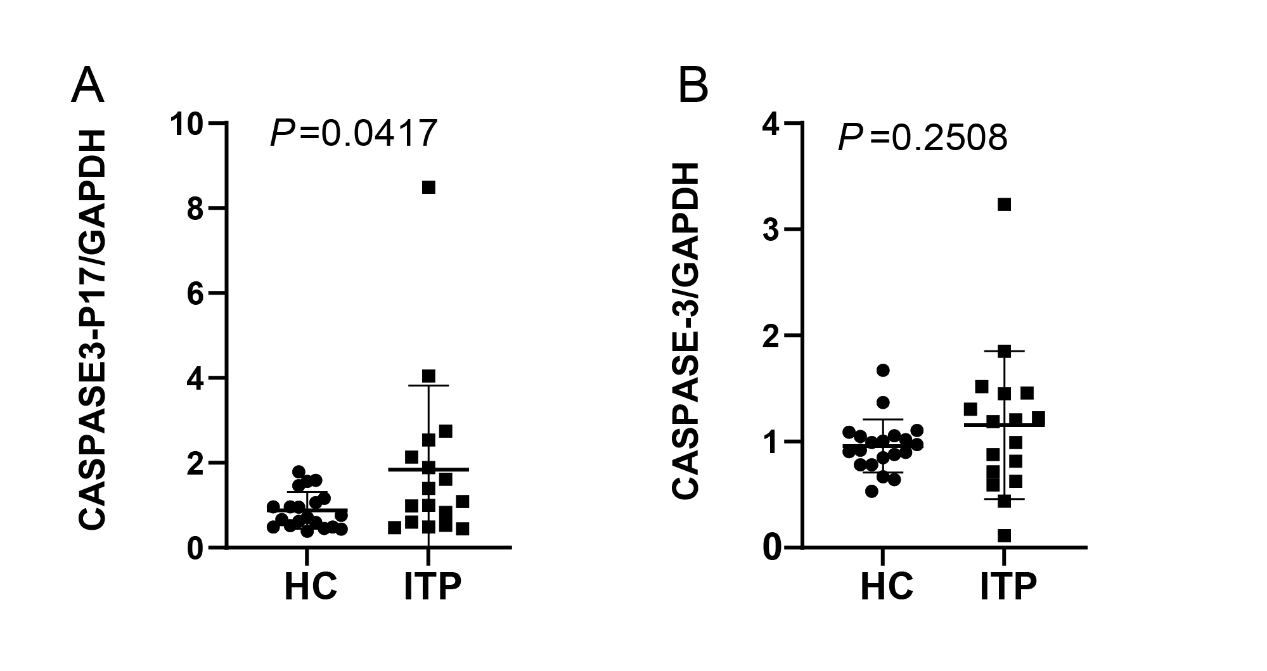


**Figure S5 The correlations of Caspases, COX-2 and platelet count in ITP patients.** (A) The correlations of platelet count with the protein expressions of Caspase-3 p17 subunit. (B) The correlations of platelet count with the protein expressions of Caspase-1. (C) The correlations of the expression of COX-2 protein levels with Caspase-3 p17 subunit. Plts: platelet count.

**
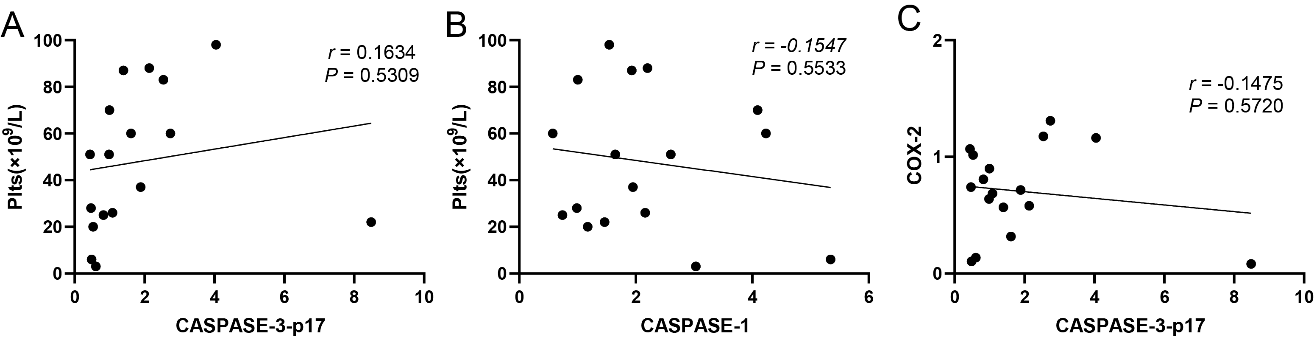
**

**Figure S6** **The impacts of COX-2 inhibitor firocoxib on megakaryopoiesis and thrombopoiesis in Meg01 cells.** Meg01 cells were treated with 100ng/ml COX-2 inhibitor firocoxib in vitro. (A) The relative count of CD41^+^CD61^+^ megakaryocytes in Meg01 cells. (B) Mean fluorescence intensity (MFI) of CD41 in CD41^+^CD61^+^ megakaryocytes. (C) Percentages of CD62P^+^ cells in CD41^+^CD61^+^ megakaryocytes. (D) Percentages of Annexin V^+^ apoptotic cells in CD41^+^CD61^+^ megakaryocytes. (E) Percentages of polyploid cells in CD41^+^CD61^+^ megakaryocytes. * *P* < 0.05, ***P* < 0.01.


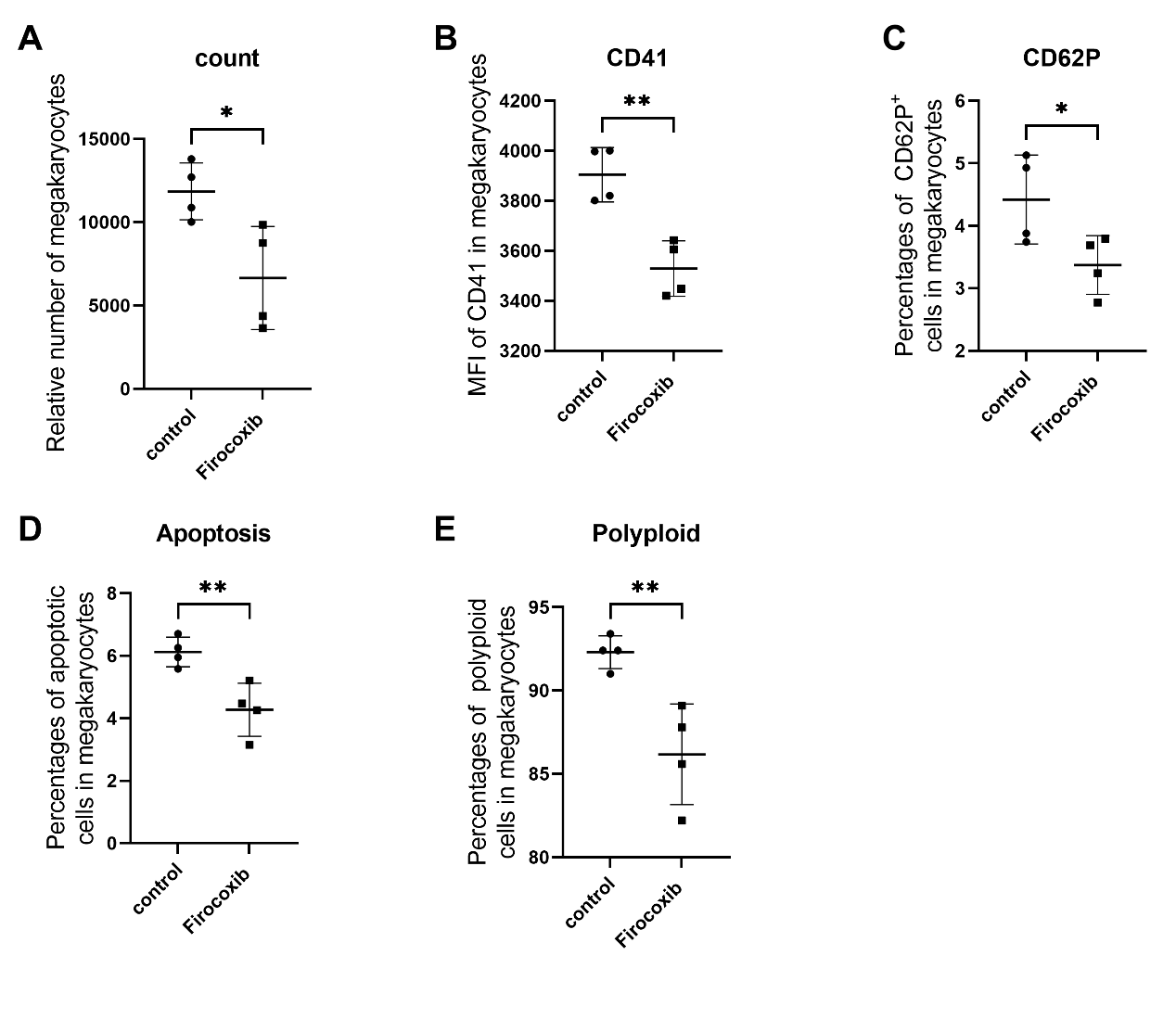

Supplement: Supplementary file 1 — Additional file 1: Table S1. Primer sequences of targeted genes. Table S2. Fluorescence channels for flow cytometry. Table S3. Correlations of CD41, COX-2 and megakaryocytes in bone marrow of ITP. Table S4. Correlations of age and gender in ITP patients. Figure S1. The consort diagram for enrolled ITP patients. Figure S2. The residual leukocytes in washed platelets flow cytometry dot plot showed the percentages of CD45+ leukocytes in washed platelets. Figure S3. The mRNA levels of residual leukocytes in washed platelets and leukocytes. The qRT-PCR results showed the mRNA expression of CD45 (PTPRC) in washed platelets and leukocytes from ITP patients and healthy controls. Plt: platelets, HC: healthy controls. ***P < 0.001. Figure S4. The expressions Caspase-3 p17 subunit and Caspases-3 in platelets of ITP patients and healthy controls. A Relative protein expression of Caspase-3 p17 subunit in platelets from ITP patients and healthy controls. B Relative protein expression of Caspase-3 in platelets from ITP patients and healthy controls. Figure S5. The correlations of Caspases, COX-2 and platelet count in ITP patients. A The correlations of platelet count with the protein expressions of Caspase-3 p17 subunit. B The correlations of platelet count with the protein expressions of Caspase-1. C The correlations of the expression of COX-2 protein levels with Caspase-3 p17 subunit. Plts: platelet count. Figure S6. The impacts of COX-2 inhibitor firocoxib on megakaryopoiesis and thrombopoiesis in Meg01 cells. Meg01 cells were treated with 100 ng/ml COX-2 inhibitor firocoxib in vitro. A The relative count of CD41+CD61+ megakaryocytes in Meg01 cells. B Mean fluorescence intensity (MFI) of CD41 in CD41+CD61+ megakaryocytes. C Percentages of CD62P+ cells in CD41+CD61+ megakaryocytes. D Percentages of Annexin V+ apoptotic cells in CD41+CD61+ megakaryocytes. E Percentages of polyploid cells in CD41+CD61+ megakaryocytes. *P < 0.05, **P < 0.01. [file 12967_2023_4389_MOESM1_ESM.docx]
